# Anterior Insula Drives Progressive Structural Brain Network Atrophy in the Behavioural Variant of Frontotemporal Dementia

**DOI:** 10.1002/hbm.70374

**Published:** 2025-10-09

**Authors:** Tao Chen, Rebekah M. Ahmed, Manisha Narasimhan, Tianyu Yang, David Foxe, Olivier Piguet, Muireann Irish

**Affiliations:** ^1^ The University of Sydney Brain and Mind Centre Sydney Australia; ^2^ The University of Sydney School of Psychology Sydney Australia; ^3^ The University of Sydney Central Clinical School Sydney Australia; ^4^ Department of Neurology The Sutherland Hospital Sydney Australia; ^5^ Zhengzhou Qianwen Intelligence Technology Co., Ltd Zhengzhou China

**Keywords:** disease progression, frontotemporal dementia, insula, network degeneration, structural covariance

## Abstract

The behavioural variant of frontotemporal dementia (bvFTD) is a younger‐onset dementia syndrome characterised by early atrophy of frontoinsular cortices, manifesting in profound socioemotional disturbances. Converging evidence from correlational, data‐driven, and computational approaches indicates large‐scale network degeneration in bvFTD. While the insula is consistently implicated, it remains unclear whether insular atrophy causally impacts progressive large‐scale structural network alterations in bvFTD. Eighty‐two patients with clinically probable bvFTD were classified as *very mild/mild* (*n* = 35), *moderate* (*n* = 30), and *severe* (*n* = 17) using the CDR plus NACC FTLD. Grey matter volume comparison between the entire bvFTD group and a healthy control group matched for age and education identified the left anterior insula as the initial maximal site of atrophy in bvFTD. To determine potential causal effects of insular atrophy on network‐based dysfunction in bvFTD, a voxel‐wise causal structural covariance network (CaSCN) was constructed based on pseudo‐time‐series morphometric data using the left anterior insula as the seed region. Sex, age, years of education, total intracranial volume (TIV), and scanning site were included as covariates, along with the difference between the sum of boxes score for the CDR plus NACC FTLD across the two pseudo–time points. Finally, an event‐based model (EBM) was applied to confirm the sequence of regional atrophy precipitated by left anterior insula atrophy, which emerged in the CaSCN analysis. BvFTD patients in the very mild/mild disease subgroup showed predominant atrophy of frontotemporal (e.g., insula, middle frontal gyrus), limbic (e.g., hippocampus, amygdala), and subcortical (e.g., putamen, nucleus accumbens) structures. Widespread grey matter atrophy was evident in the moderate bvFTD subgroup, extending to the middle cingulate, paracingulate gyri, and the thalamus, which progressed to posterior brain regions, including the fusiform gyrus and the cerebellum in the severe subgroup. Importantly, the CaSCN and event‐based model analysis reinforced the disease‐staging results by revealing progression of atrophy from the initial seed region of the left anterior insula to the orbitofrontal cortex, putamen/nucleus accumbens, anterior cingulate cortex, dorsolateral prefrontal cortex, inferior temporal gyrus, and supramarginal gyrus, before progressing posteriorly to the lingual gyrus. Using causal structural covariance network analysis and event‐based modelling, our findings indicate a causal role for the left anterior insula in driving the spread of pathology in bvFTD through well‐delineated functional brain networks known to support higher‐order cognitive and socioemotional processing. By capturing the direction of atrophy progression, our findings hold utility for potentially monitoring and tracking the efficacy of novel therapeutics on brain function in bvFTD.

## Graphical abstract

The causal structural covariance network (CaSCN) and event‐based model analysis demonstrated progression of structural atrophy in the behavioural variant of frontotemporal dementia (bvFTD) from the left anterior insula to the orbitofrontal cortex, putamen/nucleus accumbens, anterior cingulate cortex, dorsolateral prefrontal cortex, inferior temporal gyrus, and supramarginal gyrus, before progressing posteriorly to the lingual gyrus.

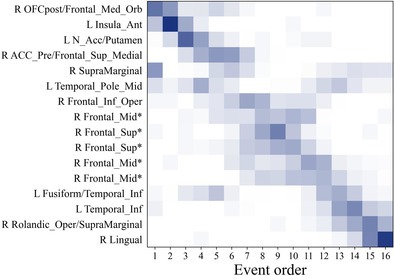

## 1 Introduction

Frontotemporal dementia (FTD) refers to a collection of younger‐onset dementia syndromes that typically strike during midlife. Advances in structural and functional neuroimaging approaches have led to the recognition that FTD syndromes rarely target discrete brain regions in isolation but progressively spread through well‐delineated large‐scale functional brain networks (Irish et al. 2012; Pievani et al. 2011). This is perhaps best exemplified by the behavioural variant of FTD (bvFTD) clinical phenotype, in which brain atrophy initially targets frontoinsular and anterior cingulate cortical regions of a putative Salience Network (Seeley 2008; Kim et al. 2011) before progressing to adjacent prefrontal and lateral temporal regions that make up the frontoparietal, default mode, and limbic networks (Eldaief et al. 2023). The progressive spread of neurodegeneration through distributed neural networks brings with it a cascade of cognitive, socioemotional, and behavioural changes with grave impacts on the individual's everyday functioning and quality of life (Rascovsky et al. 2011; Seeley et al. 2008; Ahmed et al. 2016).

Understanding how disease progression influences the propagation of atrophy through brain networks is a nascent topic in the bvFTD research literature. For example, structural MRIs have been leveraged to measure changes in grey matter atrophy across different disease stages of bvFTD. An early study by Seeley and colleagues applied voxel‐based morphometry (VBM) analysis to 45 bvFTD patients spanning three stages of disease severity based on the Clinical Dementia Rating (CDR) scale (Seeley et al. 2008). Notably, the mildest bvFTD group was associated with focal atrophy to frontoinsular and dorsal anterior cingulate cortices, key regions of the Salience Network, while posterior grey matter structures including the right cerebellum were implicated at the advanced disease stage. Importantly, these atrophy patterns were replicated in a recent investigation, which uncovered stronger and more widespread grey matter volume loss at a more advanced disease stage in bvFTD (Bejanin et al. 2020). Longitudinal studies assessing changes in cortical thickness further suggest the progressive encroachment of atrophy from cortical to subcortical regions in bvFTD (Landin‐Romero et al. 2017), while changes in resting‐state low‐frequency fluctuations within the insula show a predictive relationship with advancing disease severity in this syndrome (Day et al. 2013).

Articulating the spread of atrophy across different stages of bvFTD is crucial for understanding underlying disease pathophysiology and modelling the efficacy of new treatment approaches. Despite progress in this field, several issues warrant consideration. First, the methodology used to determine disease staging in bvFTD is a crucial determinant of the findings. In many studies (Seeley et al. 2008; Bejanin et al. 2020), bvFTD disease staging was determined using the CDR; a measure originally developed for use in Alzheimer's disease, which neglects core behavioural and language symptoms of bvFTD (Miyagawa et al. 2020). Whether other disease‐staging tools validated for use in bvFTD would produce different findings remains unclear. Secondly, voxel‐based or vertex‐based approaches focus on local grey matter or surface atrophy rather than network degeneration (Bejanin et al. 2020; Landin‐Romero et al. 2017). While informative, such approaches overlook the principle that brain disorders (e.g., neurological diseases) are, at their core, network disorders (Fornito et al. 2015; Stam 2014). Efforts to map the interregional dependence of multiple brain regions with initial regional atrophy and their relationship to disease progression in bvFTD remain lacking.

Structural covariance network analysis has been put forward as an attractive means of mapping synchronous interregional grey matter atrophy in bvFTD (Seeley et al. 2009; Hafkemeijer et al. 2016; Vogel et al. 2023). Researchers demonstrated that a frontoinsular‐associated structural covariance network maps well onto the canonical brain regions targeted in bvFTD, highlighting the value of network‐based approaches in neurodegeneration (Seeley et al. 2009). Computational models have further been used to more strictly test network‐based progression of atrophy in bvFTD. For example, a network diffusion model simulating diffusive prion‐like propagation was found to predict the atrophy pattern of bvFTD (Raj et al. 2012). More recently, a Susceptible‐Infected‐Removed (SIR) agent‐based model demonstrated that anatomical connections play a key role in shaping the canonical bvFTD atrophy pattern (Shafiei et al. 2022). Although structural covariance network analysis offers insights into network‐based degeneration (Vogel et al. 2023), the approach is zero‐time lagged, meaning it cannot quantify the temporal associations between disease epicentres and multiple impacted brain regions.

One possible solution to these challenges lies in the application of causal structural covariance network (CaSCN) analysis, which can capture the direction of information flow within distributed brain networks. Application of a Granger Causal Approach (GCA) to the pseudo‐time‐series morphometric data provides a useful tool to elucidate interregional temporal precedence of multiple atrophied regions given a known disease epicentre (Li et al. 2022; Qing et al. 2021). Specifically, if compared with the past value of time series Y alone, the combination of past value of time series X and Y can better predict the present value of time series Y, we can conclude that X has Granger causal influence on Y (Granger 1969). GCA has previously been used in neuropsychiatric disorders such as schizophrenia to explore if the neural activity of one brain region precedes that in another region using time‐series functional imaging data (Sridharan et al. 2008; Palaniyappan et al. 2013). Recently, it has been proposed that if cross‐sectional morphometric data are given temporal information regarding disease progression, GCA could be used to construct the CaSCN, thereby providing an effective approach to model atrophy progression within structural networks (Zhang et al. 2017). Indeed, CaSCN has been successfully applied to a wide variety of clinical populations including psychiatric (Han et al. 2023; Jiang et al. 2018), and neurodevelopmental (Guo, Duan, et al. 2020) disorders. Moreover, CaSCN has been deployed to explore progressive structural network atrophy in neurodegenerative disorders including Alzheimer's disease and Parkinson's disease (Li et al. 2022; Qing et al. 2021). No study, to our knowledge, has employed this technique to uncover the profile of progressive brain structural atrophy in bvFTD.

The present study therefore sought to explore the progressive structural network pattern of bvFTD using morphometric data. To rectify previous issues regarding disease staging, we examined stage‐specific grey matter volume loss in bvFTD patients as determined by the CDR Dementia Staging Instrument PLUS National Alzheimer's Coordinating Center (NACC) Behavior and Language Domains (CDR plus NACC FTLD) (Miyagawa et al. 2020; Knopman et al. 2008, 2011). In line with previous findings, we set the left anterior insula as the disease epicentre for bvFTD (Seeley et al. 2009; Shafiei et al. 2022; Agosta et al. 2023), following which whole‐brain voxel‐wise CaSCN with pseudo‐time‐series morphometric data ranked by disease severity was constructed to map anterior insula‐driven structural network atrophy in bvFTD. Importantly, while the CaSCN analysis identifies which regions are causally affected by the initial atrophy of the left anterior insula, it does not determine the sequential order in which these regions subsequently undergo atrophy. To address this issue, we employed an event‐based model to delineate the progression of atrophy among the brain regions identified by the CaSCN analysis, enhancing our ability to monitor disease progression in bvFTD.

## 2 Materials and Methods

### 2.1 Participants

A total of 82 individuals diagnosed with bvFTD and 80 healthy controls were recruited through the FRONTIER research clinic based at the Brain and Mind Centre, The University of Sydney, Australia. This sample was determined from a retrospective review of cases seen during the period of 2008–2023 that satisfied study inclusion criteria. Patients with bvFTD were included if they fulfilled current diagnostic criteria for probable or definite bvFTD (Rascovsky et al. 2011). Diagnoses were conferred by a multidisciplinary team including a neuropsychologist, senior neurologist, and occupational therapist based on neurological examination, informant report, comprehensive cognitive assessment, and structural brain imaging. Patients were excluded if they scored < 40 on the Addenbrooke's Cognitive Examination III ACE‐III; (Hsieh et al. 2013) indicating severe cognitive impairment. Non‐progressive cases of bvFTD (i.e., phenocopy cases) and cases with suspected motor involvement (i.e., FTD‐MND) were excluded.

Healthy controls were recruited from the FRONTIER volunteer database and local community groups and were required to score 88 or above on the ACE‐III (max score: 100). Exclusion criteria for all participants consisted of a history of serious mental illness, significant head injury, alcohol/substance abuse, or limited English proficiency.

### 2.2 Clinical and Cognitive Assessment

The ACE‐R (Mioshi et al. 2006) and ACE‐III (Hsieh et al. 2013) were used to measure global cognitive function across the domains of Attention and Orientation, Memory, Fluency, Language, and Visuospatial abilities. Scores from the ACE‐R were converted to ACE‐III equivalents using established algorithms (So et al. 2018). The Cambridge Behavioural Inventory‐Revised (CBI‐R) provided an index of informant‐rated behavioural changes (Wedderburn et al. 2008) while the CDR plus NACC FTLD (Sum of Boxes) was used to evaluate disease severity in bvFTD. Employing the recently validated approach to determine disease staging of frontotemporal dementia using the CDR plus NACC FTLD (Miyagawa et al. 2020) enabled us to classify bvFTD patients into the following disease stages: (very) mild (Stage 1), moderate (Stage 2), or severe (Stage 3) where higher scores reflect more advanced disease staging. Disease duration was recorded as years elapsed from reported first symptom onset to the current date of testing.

### 2.3 Statistical Analyses of Behavioural Data

Cognitive and clinical data were analyzed using JASP (Love et al. 2019). Group differences between bvFTD and control participants on continuous variables were conducted using a series of two‐sample t‐tests. A Chi‐square test was used to investigate group differences on categorical variables (e.g., sex). Where data were missing, multiple imputation was run using the MICE package in R software to obtain a complete dataset of the key cognitive and clinical variables in the study (Van Buuren and Groothuis‐Oudshoorn 2011).

### 2.4 Image Acquisition

Participants underwent whole‐brain T1‐weighted structural imaging on a 3 T MRI scanner equipped with a standard 8‐channel head coil. The T1‐weighted images were acquired using the following sequences: coronal orientation, matrix 256 × 256, 200 slices, 1 × 1 mm in‐plane resolution, slice thickness = 1 mm, echo time/repetition = 2.6/5.8 ms, flip angle of 8°. All images were subject to quality control by an experienced rater. Participants assessed until December 2016 were scanned on a 3 T Philips MRI scanner and on a 3 T GE Discovery MR750 scanner from January 2017. As such, a dummy variable to control for scanning site was included in all imaging analyses.

### 2.5 Data Preprocessing

Structural MRI data preprocessing was performed with the CAT12 morphological processing toolbox (CAT12; http://www.neuro.uni‐jena.de/cat/), embedded in Statistical Parametric Mapping software (SPM12; https://www.fil.ion.ucl.ac.uk/spm/). T1‐weighted structural images were initially reoriented to the same spatial orientation and the image origin of the anterior commissure by manual setting. Then, the standard settings of the CAT12 toolbox were applied: bias corrections for magnetic field inhomogeneity, precise segmentation into grey matter, white matter, and cerebrospinal fluid, spatial normalization to the Montreal National Institute (MNI) space according to the transformation parameters from a study‐specific DARTEL template (voxel size: 1.5 mm × 1.5 mm × 1.5 mm). Finally, the modulated, segmented images were smoothed with a 3‐mm fullwidth at half maximum isotropic Gaussian kernel. A 0.1 absolute masking threshold was applied. Automated measures of quality control were implemented in the CAT12 toolbox alongside visual inspection of the data to ensure the quality of the pre‐processed data.

### 2.6 Voxel‐Based Morphometric Analyses

First, we sought to identify overall grey matter volume atrophy within the bvFTD group relative to controls. A two‐sample *t*‐test was conducted in SPM12 to compare smoothed, modulated grey matter maps between the whole bvFTD group and the healthy control group (*p* < 0.005, FDR correction) with a cluster extent threshold of 300 contiguous voxels. Next, we sought to explore the progression of atrophy across the three disease stages derived using the CDR plus NACC FTLD (Miyagawa et al. 2020). This resulted in a stage‐specific comparison of mild (*n* = 35), moderate (*n* = 30), and severe (*n* = 17) bvFTD patients. The smoothed modulated grey matter data for each bvFTD disease‐stage subgroup was then compared to its corresponding control group via a series of two‐sample t‐tests (*p* < 0.005, FDR correction) with a cluster size of 300 contiguous voxels. For all comparisons, sex, age, years of education, total intracranial volume (TIV), and scanning site were included as covariates.

### 2.7 Seeds of Initial Atrophy

To determine the maximal site of atrophy in bvFTD, we implemented a stringent correction (*P*_FWE_ < 0.00001) and clustering (500 contiguous voxels) threshold, in keeping with previous studies (Jiang et al. 2018). This extremely conservative threshold was applied to retain only the most robust and spatially consistent effects, as an index of the regions exhibiting the earliest structural alterations in bvFTD. This analysis revealed that the peak atrophy voxel resided in the left insula (*p* < 0.00001, FWE correction) with atrophy predominantly confined to the anterior portion. Finally, using the insula atlas from Deen and colleagues, we extracted a left AI seed as the overlapping region between the surviving cluster and the left AI mask (Deen et al. 2011).

### 2.8 Voxel‐Wise Causal Structural Covariance Network Analysis

To test if the initial targeted region (i.e., left anterior insula) drives the atrophy of other brain networks in bvFTD, the smoothed modulated grey matter maps of all bvFTD patients were first sequenced from low to high according to the ranked CDR plus NACC FTLD sum‐box scores. This enabled us to attribute “pseudo‐time‐series” information to the cross‐sectional structural imaging data based on disease stage. Then, the voxel‐wise seed‐based CaSCN was constructed by applying GCA to the “pseudo‐time series” morphological data. The left anterior insula was used as the initial seed, as this region emerged as the site of greatest atrophy in the full sample comparison of bvFTD to controls (see Supplementary Figure S1). As a comparison, we conducted a separate analysis in which the right anterior insula was used as the seed to perform causal structural covariance network analysis followed by event‐based modelling (see Supplementary Figure S1–S3 and Table S3). The signed‐path coefficient of GCA was used to describe the potential progressive network atrophy caused by left anterior insular atrophy with pseudo‐time‐sequenced data. Signed‐path coefficient GCA analysis was implemented on a voxel‐wise basis within a whole brain mask using REST‐GCA, embedded in REST software (http://www.rest‐fmri.net). The positive signed‐path coefficient from region X (e.g., left anterior insula) to region Y (e.g., putamen) reflects the temporally lagged grey matter volume loss of Y following the seed atrophy of X. As such, only the positive signed‐path coefficient from X to Y (from the left anterior insula to the whole brain) was adopted given our a priori prediction regarding the causal role of the left anterior insula epicentre in bvFTD. Sex, age, years of education, TIV, scanning site, and time interval between two pseudo–time points were included as covariates in the voxel‐wide CaSCN analyses. Finally, the positive GCA map was transformed to a *z*‐score, and the results were corrected using voxel‐level FDR correction at *p* < 0.001 with a cluster size of 100 contiguous voxels.

### 2.9 The Event‐Based Model

The event‐based model (Young et al. 2014) is a generative statistical framework for modelling disease progression by estimating the probabilistic ordering of pathological events from cross‐sectional data while quantifying uncertainty in this sequence. It infers disease progression directly from the distribution of abnormalities across individuals, without requiring predefined stages and has been used across various neurodegenerative disorders, including Alzheimer's disease (Young et al. 2014; Firth et al. 2020), Parkinson's disease (Oxtoby et al. 2021), Huntington's disease (Wijeratne et al. 2018), multiple sclerosis (Eshaghi et al. 2018) and frontotemporal dementia (FTD). (Olm et al. 2023; van der Ende et al. 2021) First, we applied a kernel density estimation‐based (KDE) mixture model to estimate normal and disease distributions for the variables of interest (e.g., grey matter volume of the left anterior insula). Then, EBM was applied to determine the most likely sequence of grey matter changes. Specifically, Greedy ascent with 10,000 iterations for each of the 10 chains is used to initialize the sequence estimation, following which, Markov Chain Monte Carlo (MCMC) sampling with 500,000 iterations is used to perform the maximum likelihood estimation on the best sequence. To enhance the reliability of our results, we employed 10 repeated stratified 5‐fold cross‐validation to ensure the robustness of the event‐based model. This process involved refitting both the mixture models and the event sequence using 80% of the cohort data for training while evaluating accuracy on the held‐out 20% in each fold. With 10 repetitions of 5‐fold partitioning, a total of 50 cross‐validation models were generated to determine the final sequence. Moreover, a positional variance diagram was used to show a probabilistic representation of uncertainty in key variable ranking within the estimated sequence across the 50 cross‐validation models. A variable with a high concentration of probability in a single column indicates strong positional stability and low uncertainty in its ranking. In contrast, variables with dispersed probability densities across multiple positions exhibit high variability, reflecting greater uncertainty in their relative ordering.

### 2.10 Ethics

Ethical approval for the study was obtained from the South Eastern Sydney Local Area Health and University of New South Wales ethics committees. Informed consent, in accordance with the Declaration of Helsinki, was obtained from all participants or their person resonsible. The conditions of our ethics approval do not permit public archiving of data supporting the conclusions of this study. Readers seeking access to the data should contact the corresponding author. Access will be granted to named individuals in accordance with ethical procedures governing the reuse of sensitive data and terms of a formal data sharing agreement.

## 3 Results

### 3.1 Clinical Characterisation of bvFTD Disease Subgroups

Demographic and clinical characteristics of study participants are provided in Table 1. Groups did not differ in terms of age or years of education (*P*s > 0.05); however, a significant group difference was found for sex distribution (*p* = 0.008), reflecting a higher proportion of males in the overall bvFTD group relative to controls. Looking at the bvFTD subgroups revealed that sex distribution, age, and years of education were comparable in each subgroup to that of the corresponding control group (*P*s > 0.05). Finally, each bvFTD subgroup displayed cognitive impairments compared to controls (*P*s < 0.001). Domain scores of CDR plus NACC FTLD in bvFTD are displayed in Table 2.

**TABLE 1:** Clinical and demographic information for bvFTD patients across different disease stages in comparison with matched Control groups^a^.

Group staging	Variable	HC	bvFTD	*t*/χ^2^ ^b^	*p*
Overall group	*n*	80	82	—	—
	Sex (M:F)	34: 46	52: 30	7.11	0.008
	Age (years)	65.58 (6.80)	63.65 (7.62)	1.70	0.090
	Education (years)	13.17 (2.57)	12.59 (3.03)	1.31	0.191
	Disease duration (years)	—	5.48 (2.33)	—	—
	ACE‐III	94.95 (3.19)	73.09 (14.19)	13.45	< 0.001
(Very) Mild	*n*	35	35	—	—
	Sex (M:F)	20: 15	20: 15	0	1
	Age (years)	63.95 (8.52)	63.69 (7.95)	0.13	0.898
	Education (years)	13.51 (2.24)	13.30 (3.75)	0.28	0.780
	Disease duration (years)	—	4.27 (2.33)	—	—
	ACE‐III	94.72 (3.41)	79.22 (12.63)	7.01	< 0.001
Moderate	*n*	30	30	—	—
	Sex (M:F)	20: 10	21: 9	0.08	0.781
	Age (years)	62.50 (8.52)	62.85 (7.51)	−0.18	0.855
	Education (years)	13.51 (2.24)	13.30 (3.75)	0.16	0.873
	Disease duration (years)	—	5.80 (3.16)	—	—
	ACE‐III	94.51 (3.50)	70.40 (13.72)	9.33	< 0.001
Severe	*n*	17	17	—	—
	Sex (M:F)	10: 7	11: 6	0.13	0.724
	Age (years)	66.80 (5.67)	64.96 (7.40)	0.81	0.422
	Education (years)	11.21 (2.69)	10.87 (1.82)	0.43	0.671
	Disease duration (years)	—	7.40 (5.48)	—	—
	ACE‐III	93.99 (2.83)	65.20 (13.33)	8.72	< 0.001

Abbreviations: bvFTD, behavioural variant of frontotemporal dementia; F, Female; HC, healthy control; M, Male.

^a^ Values show means with standard deviations in parentheses.

^b^ Chi‐squared test used to test group differences on categorical variables.

**TABLE 2:** Distribution of sum of boxes and domain scores of CDR plus NACC FTLD in bvFTD.

	Overall sample (*n* = 82)	Mild stage (*n* = 35)	Moderate stage (*n* = 30)	Severe stage (*n* = 17)
Sum of boxes	9.90 (5.09)	5.46 (1.78)	10.88 (2.07)	17.29 (3.71)
Memory	1.02 (0.74)	0.64 (0.38)	1.02 (0.62)	1.79 (0.92)
Orientation	1.04 (0.73)	0.64 (0.38)	1.00 (0.53)	1.91 (0.85)
Judgment and problem solving	1.60 (0.91)	0.84 (0.47)	1.80 (0.61)	2.82 (0.39)
Community affairs	1.21 (0.77)	0.64 (0.31)	1.43 (0.61)	1.79 (0.84)
Home and hobbies	1.48 (0.87)	0.73 (0.35)	1.72 (0.61)	2.59 (0.51)
Personal care	0.99 (0.92)	0.31 (0.47)	1.17 (0.65)	2.06 (0.90)
Behaviours	1.36 (0.87)	0.79 (0.57)	1.47 (0.56)	2.35 (0.86)
Language	1.21 (0.89)	0.86 (0.63)	1.28 (0.78)	1.79 (1.20)

*Note:* Values represent means with standard deviations in parentheses. Higher scores on the CDR plus NACC FTLD denote greater disease burden/poorer function.

### 3.2 Overall Grey Matter Atrophy Pattern in bvFTD

First, we compared the aggregated bvFTD group to the HC group. Overall, a diffuse profile of grey matter atrophy was evident in bvFTD, spanning frontal (e.g., medial prefrontal, orbital frontal cortex), insular, lateral temporal (e.g., fusiform gyrus, superior temporal gyrus, middle temporal gyrus), and medial temporal (e.g., hippocampus, parahippocampal gyrus) cortices alongside occipital regions (e.g., middle occipital gyrus) and the cerebellum (e.g., Crus I/II of both cerebellar hemispheres). Subcortical structures, including the putamen and nucleus accumbens, were also significantly atrophied in bvFTD (Supplementary Table S1).

### 3.3 Progressive Stage‐Specific Grey Matter Atrophy Patterns in bvFTD

**FIGURE 1:**
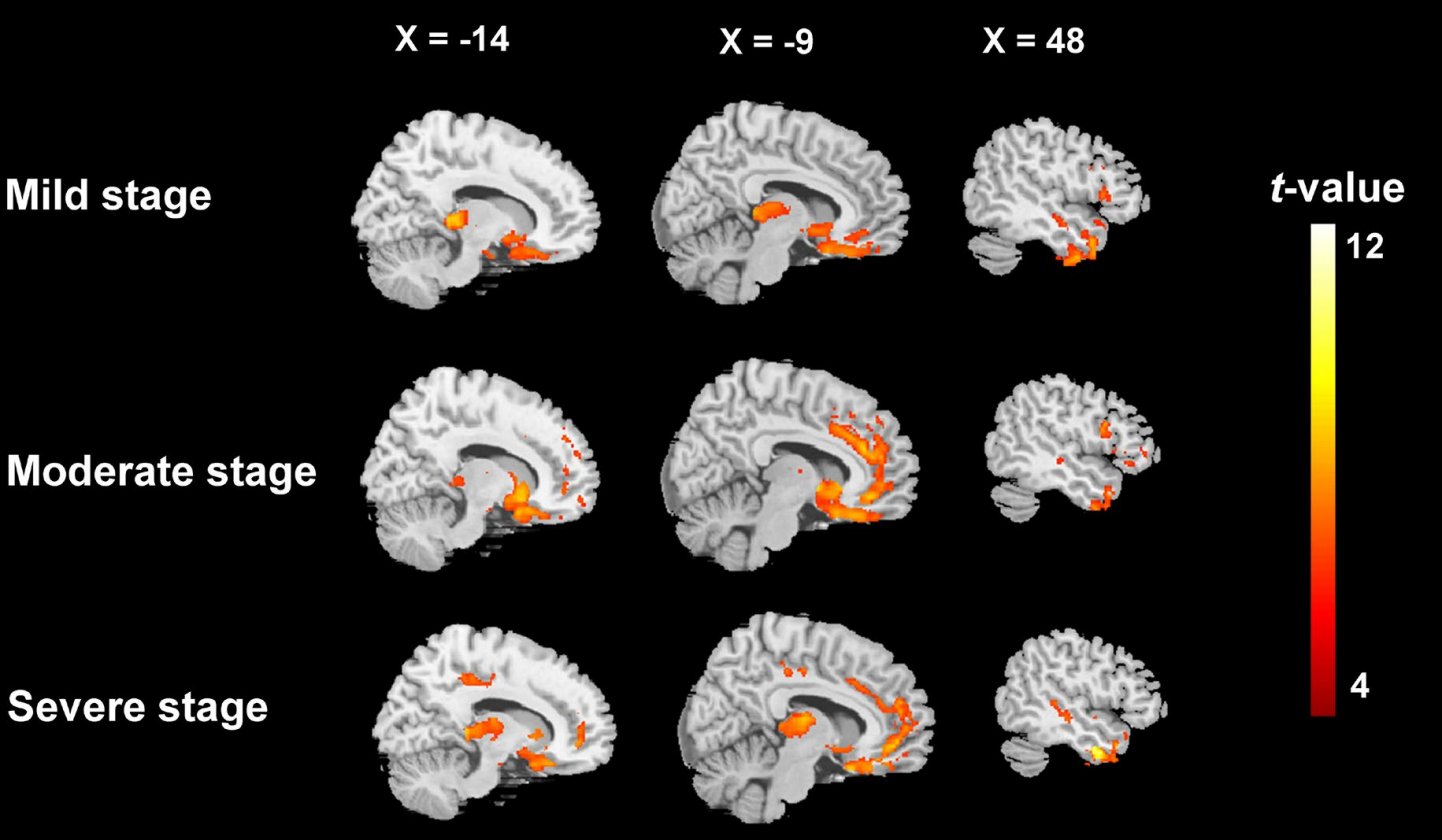
Stage‐specific profiles of grey matter atrophy in bvFTD subgroups. Disease staging determined using the CDR NACC FTLD to create (very) mild (*n* = 35), moderate (*n* = 30), and severe (*n* = 17) bvFTD subgroups. Clusters were extracted using a voxel‐level false discovery rate (FDR) correction at *p* < 0.005, with a minimum cluster extent threshold of 300 contiguous voxels. Sex, age, years of education, total intracranial volume (TIV), and scanning site were included as covariates.

Next, we considered grey matter atrophy patterns in each bvFTD subgroup relative to their respective control group. Patients in the (very) mild stage showed predominant atrophy of frontotemporal (e.g., insula, temporal pole, middle frontal gyrus, precentral gyrus), limbic (e.g., hippocampus, amygdala), and subcortical (e.g., putamen, nucleus accumbens) brain areas. In addition to the regions implicated in the (very) mild group, atrophy in the moderate disease stage included the middle cingulate cortex, paracingulate gyri, and the thalamus. Finally, the severe subgroup showed additional atrophy of posterior brain regions, including the occipital fusiform gyrus and Crus I of the cerebellum (Figure 1, Supplementary Table S2).

### 3.4 Causal Structural Covariance Network Analysis in bvFTD

**FIGURE 2:**
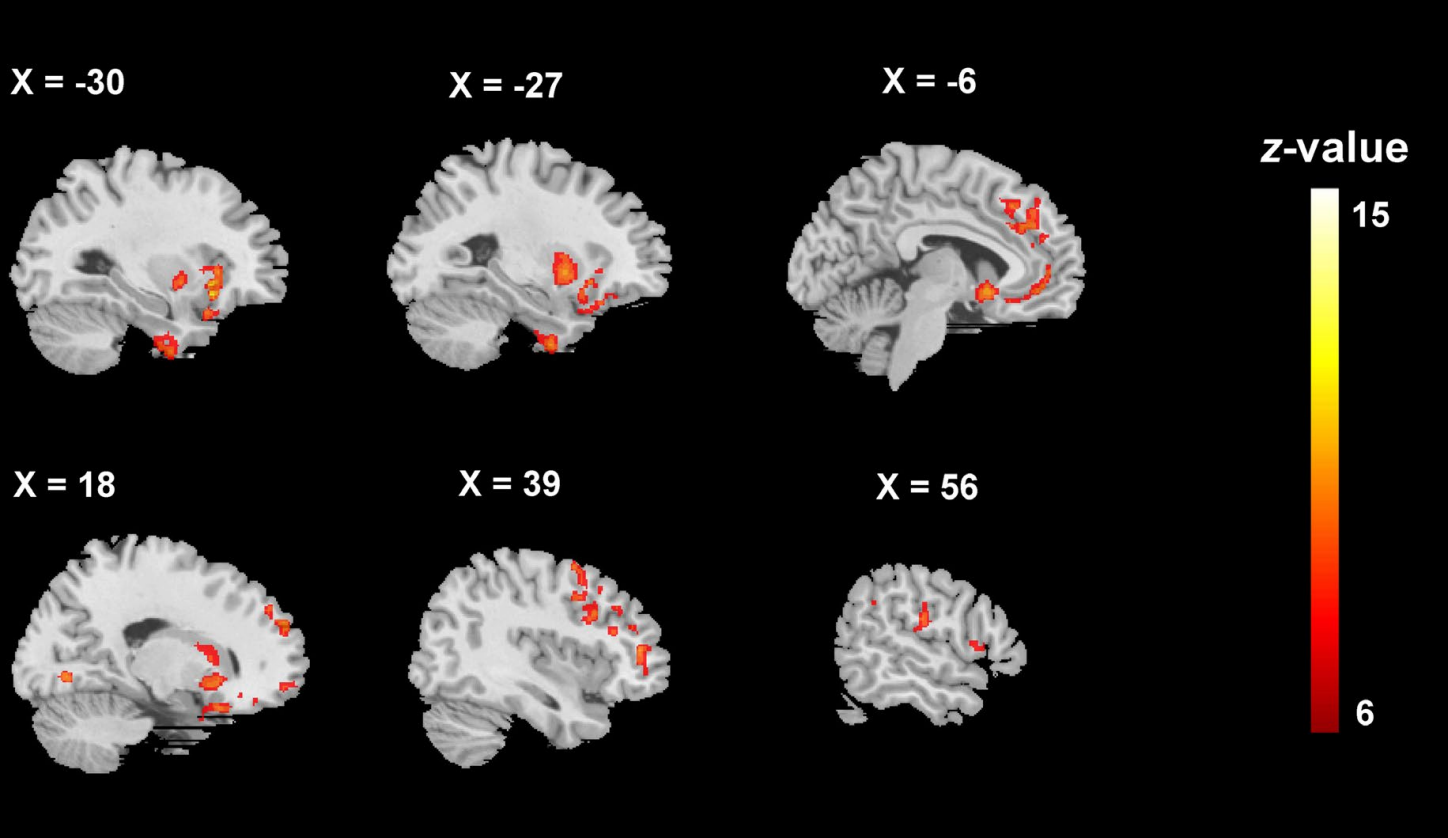
Causal effects of grey matter volume alterations across the entire bvFTD sample (*n* = 82) with the left anterior insula as the seed. Colour bar represents *z* values transformed from Granger causality values. Clusters were extracted using a voxel‐level false discovery rate (FDR) correction at *p* < 0.001, with a minimum cluster extent threshold of 100 contiguous voxels. Sex, age, years of education, total intracranial volume (TIV), scanning site, and time interval between two pseudo–time points were included as covariates.

CaSCN analysis demonstrated that grey matter atrophy progressively spread from the seed region (i.e., left anterior insula) to distinct regions within the frontoparietal network, default mode network, and the salience network, as well as some posterior brain regions, including the supramarginal gyrus and lingual gyrus (Figure 2, Table 3).

**TABLE 3:** Overview of causal effects of GMV alterations across the entire bvFTD sample with left anterior insula as the seed.

Regions	Side	Cluster size	Peak MNI coordinates			*Z*‐value
			*x*	*y*	*z*	
Left inferior temporal gyrus; left fusiform gyrus	L	519	−27	−5	−45	9.85
Left middle temporal pole; left middle temporal gyrus; left inferior temporal gyrus	L	100	−45	9	–30	6.08
Left inferior temporal gyrus	L	112	−54	−29	−26	8.87
Right medial orbital frontal gyrus; left superior medial frontal gyrus; right anterior cingulate cortex, pregenual; right caudate nucleus; left anterior cingulate cortex, pregenual; right insula; right medial orbital frontal gyrus; right superior medial frontal gyrus; right anterior cingulate cortex, subgenual; right posterior orbital frontal cortex; left medial orbital frontal gyrus; right anterior orbital frontal cortex; right olfactory cortex	R	3909	21	21	–21	13.94
Left insula; left posterior orbital frontal cortex	L	954	−30	20	–6	14.69
Right superior frontal gyrus, part 2	R	220	18	57	–9	6.61
Left putamen; left caudate nucleus; left nucleus accumbens	L	1386	−6	14	–8	10.20
Right middle frontal gyrus, part 2; right inferior frontal gyrus, orbital part 2	R	303	39	44	9	9.83
Right lingual gyrus; right fusiform gyrus	R	301	18	–72	–3	9.91
Right inferior frontal gyrus, opercular part	R	107	53	11	9	7.75
Right middle frontal gyrus, part 2; right inferior frontal gyrus, triangular part	R	593	44	30	36	8.58
Right supramarginal gyrus; right rolandic operculum; right postcentral gyrus	R	185	56	–18	23	7.98
Right superior medial frontal gyrus; right pregenual anterior cingulate cortex	R	283	9	44	29	9.66
Right superior frontal gyrus, part 2	R	225	20	57	27	10.82
Right middle frontal gyrus, part 2; right inferior frontal gyrus, opercular part	R	843	35	5	60	11.62
Right supramarginal gyrus; right angular gyrus	R	111	50	–44	33	8.62

*Note:* Clusters were extracted using a voxel‐level false discovery rate (FDR) correction at *P* < 0.001, with a minimum cluster extent threshold of 100 contiguous voxels. Sex, age, years of education, total intracranial volume (TIV), scanning site, and time interval between two pseudo–time points were included as covariates.

Abbreviations: GMV, Grey matter volume; L, Left; R, Right.

### 3.5 Sequence of Brain Atrophy in bvFTD

**FIGURE 3:**
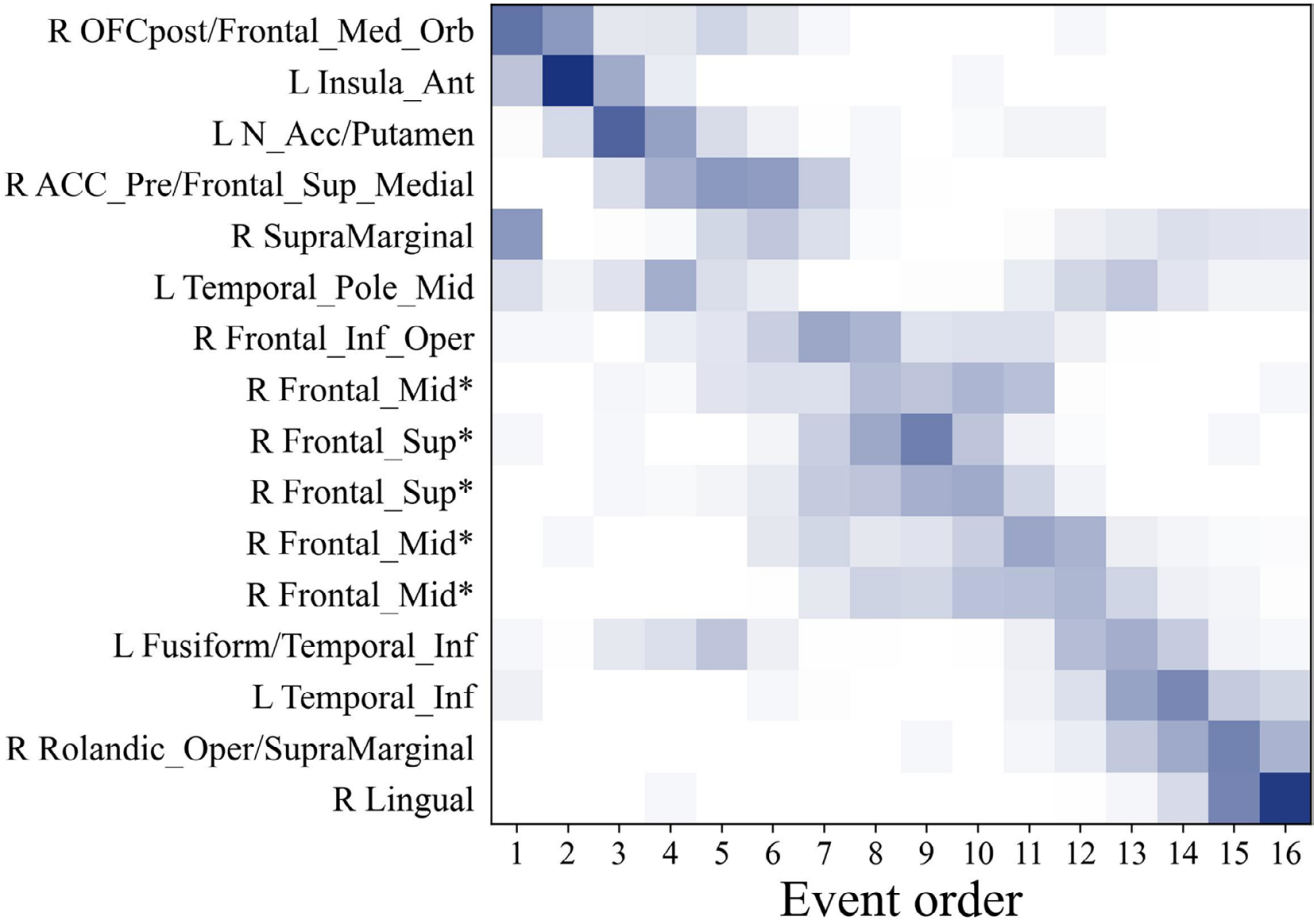
Trajectory of progressive structural atrophy originating from the left anterior insula in bvFTD. Positional variance diagram showing results of event‐based model (EBM) in bvFTD (*n* = 82) using the 16 brain regions from the causal structural covariance network analysis (CaSCN). In the positional variance diagram, colour intensity represents the certainty proportion, ranging from 0 (white) to 1 (dark blue), indicating how frequently key variables (*y*‐axis) occupy a specific position (*x*‐axis) in the event order derived from 10 repeated stratified 5‐fold cross‐validations. Fusiform/Temporal_Inf = fusiform gyrus/inferior temporal gyrus; Temporal_Pole_Mid = middle temporal pole; Temporal_Inf = inferior temporal gyrus; OFCpost/Frontal_Med_Orb = posterior orbitofrontal cortex/medial orbitofrontal cortex; Insula_Ant = anterior insula; Frontal_Sup = superior frontal gyrus; N_Acc/Putamen = nucleus accumbens/putamen; Frontal_Mid = middle frontal gyrus; Lingual = lingual gyrus; Frontal_Inf_Oper = inferior opercular frontal gyrus; Rolandic_Oper/SupraMarginal = rolandic operculum/supramarginal gyrus; ACC_Pre/Frontal_Sup_Medial = pregenual anterior cingulate cortex/medial superior frontal gyrus; SupraMarginal = supramarginal gyrus. L = Left; *R* = Right. The asterisk (*) denotes a different spatial location within the same anatomical brain region.

The brain regions affected by the atrophy of the left anterior insula seed were selected as the variables in the event‐based model (EBM). As depicted in Figure 3, cross‐validated EBM results indicated a sequential trajectory of atrophy, initiating in the left anterior insula and subsequently extending to the orbitofrontal cortex, putamen/nucleus accumbens, anterior cingulate cortex, dorsolateral prefrontal cortex, inferior temporal gyrus, and supramarginal gyrus, before ultimately progressing posteriorly to the lingual gyrus.

## 4 Discussion

Neurodegenerative disorders are characterised by widespread brain atrophy, which typically originates in focal disease epicentres and propagates through distributed brain networks. Understanding the spatial pattern of pathological spread through coordinated brain networks represents a fundamental challenge for the field (Shafiei et al. 2022; Jiang et al. 2023; Brown et al. 2019) yet is essential for accurate disease monitoring and tracking the efficacy of disease‐modifying therapeutics. Here, we used cross‐sectional disease stage‐specific comparisons, causal structural covariance network analysis (CaSCN), and event‐based modeling (EBM) to delineate the trajectory of progressive structural atrophy in bvFTD. Our cross‐sectional analysis isolated the left anterior insula as the site of maximal early changes (i.e., disease epicentre) in bvFTD (Seeley et al. 2009; Shafiei et al. 2022; Agosta et al. 2023) from which atrophy progression was predicted. Crucially, the CaSCN and EBM jointly revealed that the transmission of atrophy originating in the left anterior insula in bvFTD follows well‐delineated pathways through large‐scale functional brain networks, providing new insights into the spread of pathology in this syndrome.

Traditional stage‐specific comparisons provide valuable information regarding cross‐sectional atrophy profiles across disease stages in bvFTD but cannot inform our understanding of progressive structural atrophy from a network perspective. Leveraging CaSCN enabled us to overcome this methodological constraint by using pseudo‐time‐series morphometric data ranked by disease severity to determine the potential causal influence of left anterior insula atrophy on progressive brain network atrophy. First, we found that atrophy progressed from the left anterior insula into other nodes of the Salience Network, like the nucleus accumbens. The Salience Network plays a crucial role in the detection of behaviourally relevant stimuli in the external environment and the initiation of switching between the default mode network, focused on internally driven cognition, and the executive control network, which supports externally oriented attention and monitoring (Menon 2011). Degeneration of the Salience Network has long been implicated in the origin of socioemotional and behavioural disturbances in bvFTD (Seeley 2008) with the insula proposed as a key hub in mediating these changes (Kumfor et al. 2015; Dermody et al. 2016).

Our CaSCN findings resonate well with the integrated network hub view of the insula (Uddin et al. 2017), given that atrophy was found to progress from the anterior insula disease epicentre to key regions within attentional networks including the FPCN (e.g., DLPFC) and hence to key regions of the DMN (e.g., medial prefrontal cortex), the orbitofrontal cortex, striatum (e.g., putamen, caudate, nucleus accumbens), as well as the supramarginal gyrus, lingual gyrus. Current theoretical frameworks view the insula as an integration hub linking large‐scale brain systems (Uddin et al. 2017), given its unique anatomical position and dense connections to an extensive network of cortical and subcortical brain regions. For example, empirical evidence reveals structural connections between anterior insula and anterior frontal regions including the DLPFC, mPFC, orbitofrontal cortex, ACC (Ghaziri et al. 2015) as well as subcortical regions in the striatum including the ventral putamen, caudate nucleus, and nucleus accumbens (Ghaziri et al. 2018). The dorsal anterior insula is functionally associated with fronto‐parietal association cortices (e.g., DLPFC) as well as the dorsal striatum, while the ventral anterior insula is functionally connected to orbitofrontal, ventral striatal, and subgenual ACC regions (Zhao et al. 2022). Our progressive atrophy modelling suggests that atrophy in bvFTD is likely to move anteriorly as well as posteriorly, particularly in more advanced disease stages, with implications for the emergence of clinical features at different disease timepoints.

As noted, while the CaSCN analysis identifies brain regions causally affected by the initial atrophy of the left anterior insula, it does not determine the sequential progression of atrophy across these regions. Leveraging EBM enabled us to address this issue by delineating the temporal trajectory of atrophy among the key brain regions identified by the CaSCN in bvFTD. Notably, the sequential pattern of atrophy derived from the EBM was highly consistent with our stage‐specific analysis of grey matter atrophy based on CDR plus NACC FTLD, which demonstrates a general progression from anterior to middle and, ultimately, posterior brain regions. The convergence of findings across distinct analytical frameworks reinforces the robustness of our results and can potentially account for some discrepancies in the literature. For example, Irwin et al. (Irwin et al. 2016; Brettschneider et al. 2014) identified the insula as being atrophied in the first stage of disease in bvFTD (Irwin et al. 2016), whereas Brettschneider et al. (Brettschneider et al. 2014) reported the orbitofrontal cortex as the initial region affected, followed by the insula. Our findings suggest that different subregions of the anterior insula may become atrophied at different time points, either before or after the orbitofrontal cortex; however, future longitudinal studies will be required to validate this proposal.

While our study offers novel insights into the progression of atrophy in bvFTD, several methodological considerations warrant discussion. First, it remains unclear how to precisely identify the initial locus of degeneration in bvFTD, as different approaches yield variable results (Vogel et al. 2023). The typical approach defines initial brain atrophy as the group averaged maximal atrophy for patients, irrespective of disease staging (Seeley et al. 2009; Jiang et al. 2018) while others operationalise it as the maximal atrophy of patients at the earliest disease stage (e.g., premanifest stage) (Guo, Chen, et al. 2020). Using the patient group averaged maximal atrophy, we found the left anterior insula was the maximal site of atrophy for bvFTD patients at the initial disease stage, converging well with previous reports (Shafiei et al. 2022). An important point to note, however, is that bvFTD is clinically and pathologically heterogeneous, with mounting evidence pointing to the existence of multiple subtypes (Ranasinghe et al. 2016; Whitwell et al. 2009). In addition, we observed greater atrophy in the moderate group compared to the severe group. This effect may reflect differences in sample size, as the moderate group included more participants and may have resulted in greater power to detect group differences. Future studies with larger samples stratified by disease subtype, genetic status, and underlying pathology are needed to clarify these issues as well as considering whether such patterns would bear out differently when split by sex (Irish 2025).

Finally, the sensitivity of our CaSCN should be considered given that not all regions with known functional and structural connections to the anterior insula (Ghaziri et al. 2015, 2018; Zhao et al. 2022), displayed progressive structural atrophy. Such selective disruptions may reflect local transcriptomic vulnerability to bvFTD pathology, regulating disease propagation along the connectome (Shafiei et al. 2022), however, further research is required to test this proposal. Future studies using independent datasets are needed to replicate our findings. Given that the pseudo‐time series data cannot directly indicate the true temporal sequence of disease progression, longitudinal studies, ideally incorporating pathological confirmation postmortem, will be imperative to clarify the causal morphological changes in bvFTD. Finally, given that the CaSCN and event‐based model was performed at the group level, it was not possible for us to explore brain atrophy patterns for individual cases. Thus, longitudinal studies incorporating MRI at multiple time points are needed to bridge this gap.

## 5 Conclusion

In summary, our disease stage‐specific comparison indicates the encroachment of grey matter atrophy commencing from predominantly frontoinsular brain regions towards subcortical and posterior cortices and the cerebellum. Our findings demonstrate the potential causal influence of anterior insula atrophy on large‐scale brain network degeneration, including key nodes of the Salience Network, Default Mode Network, and Frontoparietal Control Network, as well as more posterior brain regions. Collectively, our findings support the utility of CDR plus NACC FTLD in classifying disease stages in bvFTD and highlight the potential of using CaSCN analysis and EBM to predict the progression of network atrophy in bvFTD. We highlight the potential of these findings in monitoring disease progression and evaluating the efficacy of emerging therapeutic interventions in bvFTD.

## Conflicts of Interest

The authors declare no conflicts of interest.

## Supporting information


**Figure S1:** Localisation of the seeds in the left and right anterior insula in the full bvFTD sample (*n* = 82).
**Figure S2:** Causal effects of grey matter volume alterations across the entire bvFTD sample (*n* = 82) with right anterior insula as the seed.
**Figure S3:** Trajectory of progressive structural atrophy originating in the right anterior insula in bvFTD.
**Table S1:** Grey matter volume alterations in the full bvFTD sample compared to Controls.
**Table S2:** Subgroup‐specific changes in grey matter volume based on disease severity in bvFTD.
**Table S3:** Overview of causal effects of GMV alterations across the bvFTD cohort using the right anterior insula as the seed.

## Data Availability

The data that support the findings of this study are available on request from the corresponding author. The data are not publicly available due to privacy or ethical restrictions.
