# Supplementary material for: Anterior Insula Drives Progressive Structural Brain Network Atrophy in the Behavioural Variant of Frontotemporal Dementia
Source: Hum Brain Mapp. 2025 Oct 9;46(14):e70374. doi: 10.1002/hbm.70374 (PMC12509179; doi:10.1002/hbm.70374)
Supplement: Supplementary file 1 — Figure S1: Localisation of the seeds in the left and right anterior insula in the full bvFTD sample (n = 82). Figure S2: Causal effects of grey matter volume alterations across the entire bvFTD sample (n = 82) with right anterior insula as the seed. Figure S3: Trajectory of progressive structural atrophy originating in the right anterior insula in bvFTD. Table S1: Grey matter volume alterations in the full bvFTD sample compared to Controls. Table S2: Subgroup‐specific changes in grey matter volume based on disease severity in bvFTD. Table S3: Overview of causal effects of GMV alterations across the bvFTD cohort using the right anterior insula as the seed. [file HBM-46-e70374-s001.docx]

**Anterior insula drives progressive structural brain network atrophy in the behavioural variant of frontotemporal dementia**

Tao Chen^1,2,^ Rebekah M. Ahmed^1,3^, Manisha Narasimhan^1,4^, Tianyu Yang^5^, David Foxe^1,2^, Olivier Piguet^1,2^, Muireann Irish^1,2^*

^1^The University of Sydney, Brain and Mind Centre, Sydney, Australia

^2^The University of Sydney, School of Psychology, Sydney, Australia

^3^The University of Sydney, Central Clinical School, Sydney, Australia

^4^Department of Neurology, The Sutherland Hospital, Sydney, Australia.

^5^Zhengzhou Qianwen Intelligence Technology Co., LTD, Zhengzhou, China

**Correspondence to**: Muireann Irish, The University of Sydney, Brain and Mind Centre, Camperdown, NSW 2050, Sydney, Australia

**E-mail:** [muireann.irish@sydney.edu.au](mailto:muireann.irish@sydney.edu.au)

**Supplementary material includes the following materials:**

**Figure S1.** Localisation of the seeds in the left and right anterior insula in the full bvFTD sample (n = 82).

**Figure S2.** Causal effects of grey matter volume alterations across the entire bvFTD sample (n = 82) with right anterior insula as the seed.

**Figure S3.** Trajectory of progressive structural atrophy originating in the right anterior insula in bvFTD.

**Table S1.** Grey matter volume alterations in the full bvFTD sample compared to Controls.

**Table S2**. Subgroup-specific changes in grey matter volume based on disease severity in bvFTD.

**Table S3**. Overview of causal effects of GMV alterations across the bvFTD cohort using the right anterior insula as the seed.


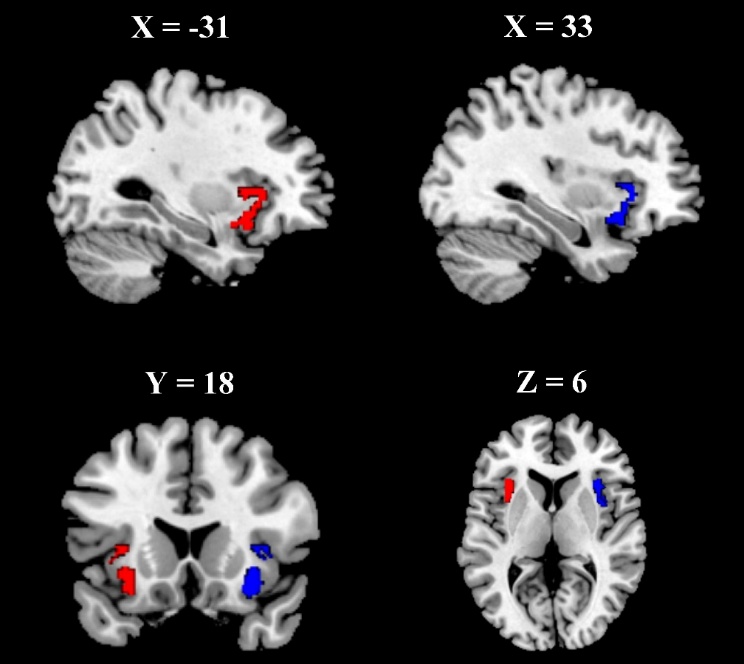


**Figure S1.** **Localisation of the seeds in the left and right anterior insula in the bvFTD sample (n = 82).** The left anterior insula seed (shown in red) corresponds to the overlapping region between the sole surviving cluster after correction (*P* < 0.00001, FWE correction) and the left anterior insula mask derived from the insula atlas by Deen et al. (2011). The right anterior insula seed (shown in blue) corresponds to the overlapping region between the solo surviving cluster (*P* < 0.00001, FWE correction, k = 500) and the right anterior insula mask from the insula atlas.


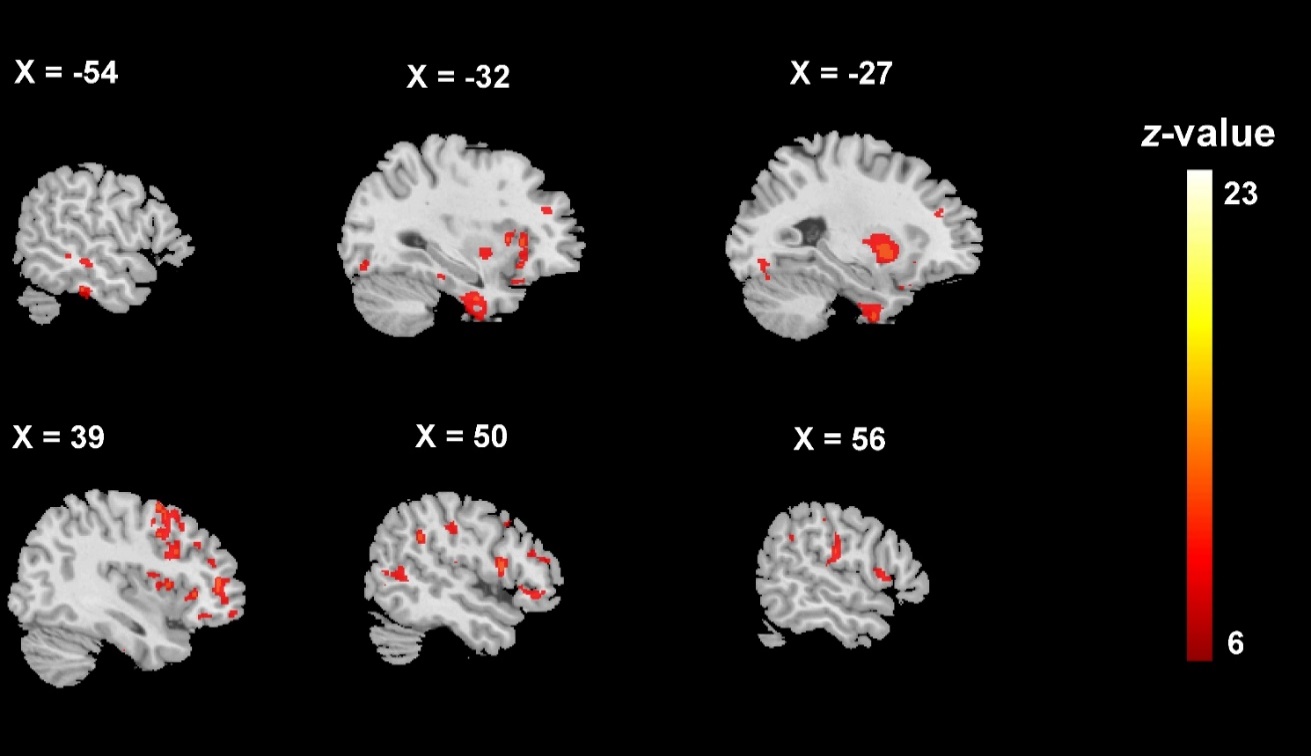


**Figure S2.** **Causal effects of grey matter volume alterations across the entire bvFTD sample (n = 82) with the right anterior insula as the seed.** Colour bar represents z values transformed from Granger causality values. Clusters were extracted using a voxel-level false discovery rate (FDR) correction at *P* < 0.001, with a minimum cluster extent threshold of 100 contiguous voxels. Sex, age, years of education, TIV, scanning site and time interval between two pseudo–time points were included as covariates.


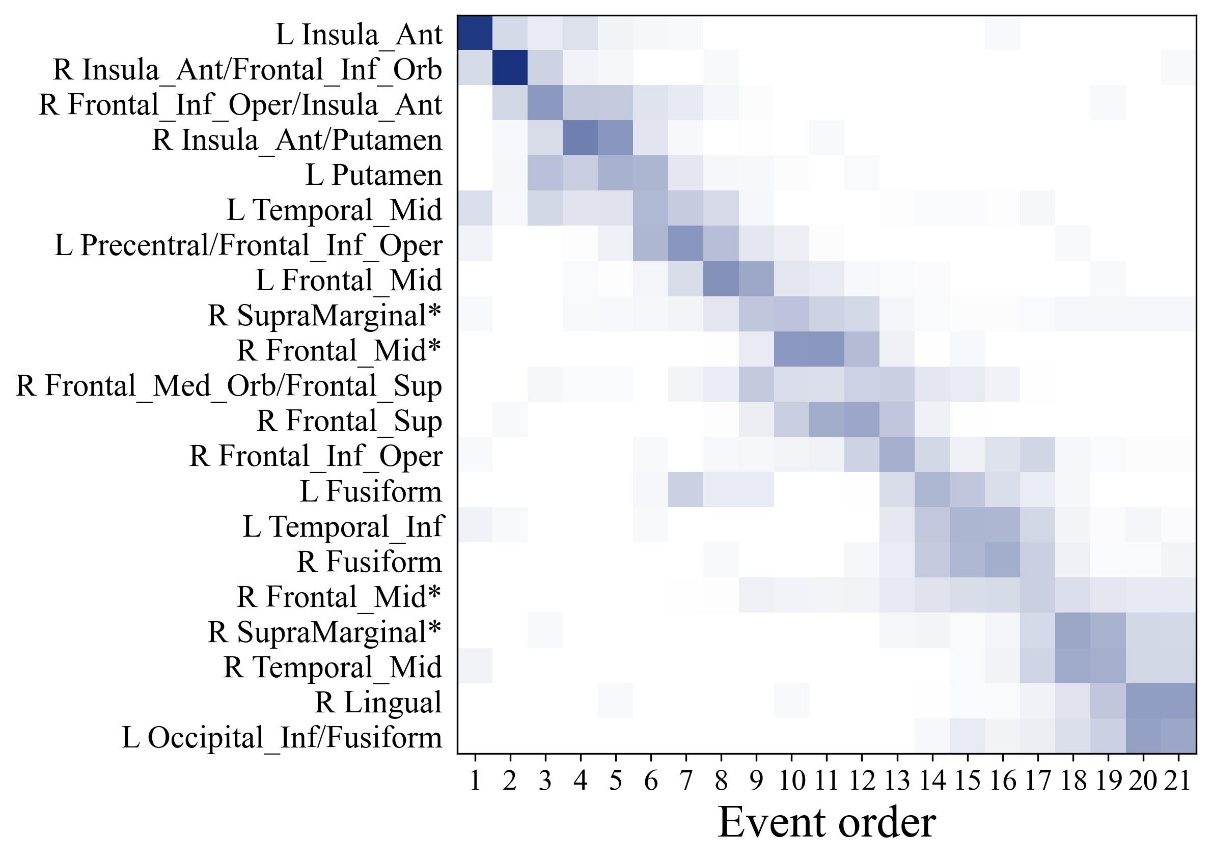


**Figure S3.** **Trajectory of progressive structural atrophy originating in the right anterior insula in bvFTD.** Positional variance diagram showing results of event-based model (EBM) in bvFTD (n = 82) using the 21 brain regions from the Causal Structural Network analysis (CaSCN). In the positional variance diagram, colour intensity represents the certainty proportion, ranging from 0 (white) to 1 (dark blue), indicating how frequently key variables (y-axis) occupy a specific position (x-axis) in the event order derived from 10 repeated stratified 5-fold cross-validations. Fusiform = fusiform gyrus; Temporal_Inf = inferior temporal gyrus; Insula_Ant/Putamen = anterior insula / putamen; Frontal_Med_Orb/Frontal_Sup = medial orbitofrontal cortex / superior frontal gyrus; Occipital_Inf/Fusiform = inferior occipital gyrus / fusiform gyrus; Putamen = putamen; Insula_Ant = anterior insula; Insula_Ant/Frontal_Inf_Orb = anterior insula / inferior orbitofrontal gyrus; Temporal_Mid = middle temporal gyrus; Lingual = lingual gyrus; Frontal_Inf_Oper/Insula_Ant = inferior opercular frontal gyrus / anterior insula; Frontal_Inf_Oper = inferior opercular frontal gyrus; Frontal_Mid = middle frontal gyrus; Frontal_Sup = superior frontal gyrus; SupraMarginal = supramarginal gyrus; Precentral/Frontal_Inf_Oper = precentral gyrus / inferior opercular frontal gyrus. L = Left; R = Right. Asterisks (*) denote a different spatial location within the same anatomical brain region.

**Table S1.** Grey matter volume alterations in the full bvFTD sample compared to Controls.

| **Regions** | **Side** | **Cluster size** | **Peak MNI coordinates** | | ***T*-value** |
| --- | --- | --- | --- | --- | --- |
|  |  |  | **x y z** |  | |
| Left insula; left middle temporal gyrus, part 1; right superior frontal gyrus, part 2; right middle frontal gyrus, part 2; left middle frontal gyrus, part 2; left superior frontal gyrus, part 2; left inferior temporal gyrus; right middle temporal gyrus, part 1; right middle cingulate gyrus; right inferior temporal gyrus; right insula; left superior frontal gyrus, medial; left middle cingulate gyrus; left inferior frontal gyrus, triangular part; right fusiform gyrus; right parahippocampal gyrus; left hippocampus; right inferior frontal gyrus, triangular part; left fusiform gyrus; right hippocampus; left parahippocampal gyrus; left superior temporal gyrus; left supplementary motor area; right superior temporal gyrus; right superior temporal pole; left putamen; right superior frontal gyrus, medial orbital; left posterior orbital gyrus; right inferior frontal gyrus, opercular part; left anterior cingulate cortex, pregenual; left inferior frontal gyrus, orbital part 2; left medial orbital gyrus; left superior frontal gyrus, medial orbital; right posterior orbital gyrus; left anterior orbital gyrus; left precentral gyrus; left superior temporal pole; right rectus gyrus; right rolandic operculum; left olfactory cortex; right putamen; right anterior cingulate cortex, pregenual; right anterior cingulate cortex, superior; right amygdala; left middle temporal pole; right medial orbital gyrus; right anterior orbital gyrus; left thalamus, pulvinar medial; left rolandic operculum; left lateral orbital gyrus; right thalamus, ventral lateral; left thalamus, ventral lateral; right thalamus, pulvinar medial; left thalamus, mediodorsal medial magnocellular; right thalamus, mediodorsal medial magnocellular; left anterior cingulate cortex, superior; right precentral gyrus; left nucleus accumbens; right posterior cingulate gyrus; right lateral orbital gyrus; left heschl gyrus; right supramarginal gyrus; right heschl gyrus; right nucleus accumbens. | Both | 88340 | -35 20 6 | 12.11 | |
| Right cerebellum crus I; right cerebellum crus II; right cerebellum lobule VI | R | 3001 | 27 -78 -38 | 6.85 | |
| Left cerebellum crus I; left cerebellum crus II; left cerebellum lobule VI | L | 2418 | -32 -72 -41 | 6.84 | |
| Right middle occipital gyrus; right angular gyrus | R | 1331 | 44 -81 9 | 5.07 | |
| Left middle occipital gyrus | L | 448 | -33 -92 3 | 5.04 | |

*Notes:* Statistical maps were thresholded at *P* < 0.005, false discovery rate (FDR) corrected at the voxel level, with a cluster extent threshold of 300 contiguous voxels. Sex, age, years of education, total intracranial volume (TIV), and scanning site were included as covariates in the model. L = Left; R = Right.

**Table S2**. Subgroup-specific changes in grey matter volume based on disease severity in bvFTD.

| **Stage** | **Regions** | **Side** | **Cluster size** | **Peak MNI coordinates** | ***T*-value** |
| --- | --- | --- | --- | --- | --- |
|  |  |  |  | **x y z** |  |
| Mild stage | Left insula, left inferior temporal gyrus; right inferior temporal gyrus; left hippocampus; right hippocampus; left middle temporal gyrus; left rectus gyrus; right middle temporal pole; right insula; left middle temporal pole; left putamen; right parahippocampal gyrus; left parahippocampal gyrus; right middle temporal gyrus; right rectus gyrus; left superior temporal pole; right fusiform gyrus; right superior temporal pole; left fusiform gyrus; left medial orbitofrontal cortex; left posterior orbitofrontal cortex; right inferior frontal gyrus, pars triangularis; right medial orbitofrontal cortex; left olfactory cortex; left medial pulvinar nucleus of thalamus; left nucleus accumbens; right olfactory cortex; right middle cingulate gyrus; right putamen; right superior anterior cingulate cortex; left medial orbitofrontal cortex; right nucleus accumbens; right inferior frontal gyrus, pars opercularis; right pregenual anterior cingulate cortex; right superior temporal gyrus; right posterior orbitofrontal cortex; left inferior frontal gyrus, pars triangularis; left inferior frontal gyrus, pars orbitalis, part 2; right inferior frontal gyrus, pars orbitalis, part 2; right anterior orbitofrontal cortex | Both | 28200 | -38 17 3 | 7.46 |
|  | Right inferior frontal gyrus, opercular part | R | 399 | 42 9 33 | 6.47 |
|  | Left precentral gyrus; left inferior frontal gyrus, opercular | L | 521 | -41 6 32 | 6.07 |
|  | Right superior frontal gyrus, part 2 | R | 335 | 23 54 26 | 5.77 |
|  | Right superior frontal gyrus, part 2; right middle frontal gyrus, part 2 | R | 367 | 24 60 -6 | 5.54 |
|  | Left superior frontal gyrus, part 2; left middle frontal gyrus, part 2 | L | 487 | -24 3 50 | 5.37 |
| Moderate stage | Right caudate; right superior frontal gyrus, part 2; left insula; right insula; right middle frontal gyrus, part 2; left putamen; right putamen; right hippocampus; left inferior frontal gyrus, triangular part; left hippocampus; right parahippocampal gyrus; left superior frontal gyrus, part 2; left rectus gyrus; right rectus gyrus; right inferior frontal gyrus, opercular part; right medial orbital gyrus; left superior medial frontal gyrus; left posterior orbital gyrus; right medial orbital gyrus; right middle temporal gyrus; left medial orbital gyrus; left anterior cingulate cortex, pregenual; right anterior cingulate cortex, pregenual; right anterior cingulate cortex, supracallosal; left anterior cingulate cortex, supracallosal; right middle cingulate cortex; right inferior temporal gyrus; left inferior orbital gyrus, part 2; left anterior orbital gyrus; right inferior orbital gyrus, part 2; right amygdala; left medial orbital gyrus; left olfactory cortex; right amygdala; right superior medial frontal gyrus; right posterior orbital gyrus; left inferior frontal gyrus, opercular part; left temporal inferior gyrus; right nucleus accumbens; left nucleus accumbens; left precentral gyrus; left middle cingulate cortex; right olfactory cortex; left supplementary motor area; right superior temporal gyrus, pole; left lateral orbital gyrus; left caudate; left superior temporal gyrus, pole; right fusiform gyrus; left fusiform gyrus; right thalamus, medial pulvinar; left middle temporal gyrus, pole | Both | 36631 | 14 20 -9 | 9.07 |
|  | Right mediodorsal thalamus, medial part; right ventrolateral thalamus | R | 895 | 3 -6 9 | 6.50 |
|  | Left middle frontal gyrus, part 2; left superior frontal gyrus, part 2 | L | 399 | -26 17 45 | 5.39 |
|  | Left middle cingulate gyrus | L | 344 | -12 -18 44 | 5.08 |
|  | Left middle temporal gyrus | L | 369 | -56 -38 -5 | 4.67 |
| Severe stage | Left insula; right insula; left hippocampus; right inferior temporal gyrus; right hippocampus; right parahippocampal gyrus; right middle temporal pole; left rectus gyrus; left putamen; right superior temporal pole; right fusiform gyrus; left superior medial frontal gyrus; right putamen; left medial orbital frontal gyrus; right middle temporal gyrus; right ventral lateral nucleus of thalamus; left medial pulvinar of thalamus; right medial pulvinar of thalamus; left posterior orbital gyrus; left anterior orbital gyrus; left medial dorsal nucleus of thalamus; left parahippocampal gyrus; right medial orbital frontal gyrus; left medial orbital frontal gyrus; left olfactory cortex; left anterior cingulate gyrus, pregenual part; left ventral lateral nucleus of thalamus; right inferior frontal gyrus, pars orbitalis, part 2; right rectus gyrus; left inferior frontal gyrus, pars triangularis; left superior temporal gyrus; left amygdala; right posterior orbital gyrus; left nucleus accumbens; right medial dorsal nucleus of thalamus; right olfactory cortex; right superior temporal gyrus; left inferior frontal gyrus, pars orbitalis, part 2; left fusiform gyrus | Both | 21655 | -36 17 3 | 11.65 |
|  | Left inferior temporal gyrus; left fusiform gyrus | L | 806 | -39 -15 -39 | 8.01 |
|  | Left middle cingulate gyrus; right middle cingulate gyrus; right pregenual anterior cingulate gyrus; right supracallosal anterior cingulate gyrus | Both | 1445 | 14 41 9 | 7.60 |
|  | Left inferior frontal gyrus, opercular part; left inferior frontal gyrus, triangular part; left precentral gyrus | L | 669 | -38 6 32 | 6.87 |
|  | Left superior frontal gyrus, part 2; left middle frontal gyrus, part 2 | L | 327 | -27 5 51 | 6.40 |
|  | Right middle temporal gyrus; right superior temporal gyrus | R | 476 | 65 -23 -6 | 5.84 |
|  | Right cerebellum crus I; right cerebellum lobule VI | R | 355 | 35 -65 -30 | 4.76 |

*Notes:* Statistical maps were thresholded at *P* < 0.005, false discovery rate (FDR) corrected at the voxel level, with a cluster extent threshold of 300 contiguous voxels. Sex, age, years of education, total intracranial volume (TIV), and scanning site were included as covariates. Disease staging determined using the CDR NACC FTLD to create (very)mild (n = 35), moderate (n = 30) and severe (n = 17) bvFTD subgroups. BvFTD = behavioural variant of frontotemporal dementia. L = Left; R = Right.

**Table S3**. Overview of causal effects of GMV alterations across the bvFTD cohort using the right anterior insula as the seed.

| **Regions** | **Side** | **Cluster size** | **Peak MNI coordinates** | | **Z-value** |
| --- | --- | --- | --- | --- | --- |
|  |  |  | **x y z** |  | |
| Left fusiform gyrus; left inferior temporal gyrus | L | 605 | -27 -5 -45 | 8.24 | |
| Right fusiform gyrus; right parahippocampal gyrus | R | 168 | 30 -6 -35 | 5.94 | |
| Left inferior temporal gyrus | L | 145 | -54 -29 -26 | 8.19 | |
| Right putamen; left superior medial frontal gyrus; right anterior cingulate cortex, pregenual part; right medial orbital gyrus; left anterior cingulate cortex, pregenual part; right caudate nucleus; right superior anterior cingulate cortex; right superior medial frontal gyrus; left medial orbital gyrus; right insula; right middle cingulate cortex; right medial orbital sulcus; right anterior orbital gyrus; right posterior orbital gyrus; right olfactory cortex; right nucleus accumbens; right anterior cingulate cortex, subgenual part | R | 6770 | 32 17 -9 | 22.71 | |
| Right superior frontal gyrus, part 2; right medial orbital gyrus | R | 501 | 5 57 -11 | 8.64 | |
| Left fusiform gyrus; left inferior occipital gyrus | L | 106 | -32 -81 -12 | 7.43 | |
| Left putamen; left caudate nucleus; left nucleus accumbens | L | 1591 | -27 3 -2 | 10.18 | |
| Left insula; left posterior orbital gyrus | L | 576 | -33 14 6 | 9.65 | |
| Right inferior frontal gyrus, orbital part, part 2; right middle frontal gyrus; right insula; right inferior frontal gyrus, triangular part | R | 627 | 35 27 5 | 10.83 | |
| Left middle temporal gyrus | L | 219 | -57 -26 -8 | 6.90 | |
| Right lingual gyrus; right fusiform gyrus | R | 390 | 17 -72 -5 | 10.34 | |
| Right insula; right inferior frontal gyrus, opercular part | R | 233 | 39 12 8 | 9.59 | |
| Right middle temporal gyrus | R | 358 | 42 -65 8 | 7.89 | |
| Right inferior frontal gyrus, opercular part | R | 216 | 51 11 9 | 11.18 | |
| Right middle frontal gyrus, part 2 | R | 353 | 45 38 29 | 8.04 | |
| Right superior frontal gyrus, part 2 | R | 409 | 20 54 30 | 10.32 | |
| Right supramarginal gyrus; right postcentral gyrus | R | 239 | 56 -18 24 | 7.39 | |
| Left inferior frontal gyrus, opercular part; left precentral gyrus; left middle frontal gyrus, part 2 | L | 320 | -38 9 30 | 7.45 | |
| Right middle frontal gyrus, part 2; right inferior frontal gyrus, opercular part | R | 1101 | 35 5 60 | 11.70 | |
| Right supramarginal gyrus | R | 114 | 50 -44 32 | 8.20 | |
| Left middle frontal gyrus, part 2; left superior frontal gyrus; left superior frontal gyrus, part 2 | L | 124 | -32 39 24 | 6.75 | |

*Note:* Clusters were extracted using a voxel-level false discovery rate (FDR) correction at *P* < 0.001, with a minimum cluster extent threshold of 100 contiguous voxels. Sex, age, years of education, TIV, scanning site and time interval between two pseudo–time points were included as covariates. GMV = Grey matter volume. L = Left; R = Right.
